# Effect of Paired-Pulse Electrical Stimulation on the Activity of Cortical Circuits

**DOI:** 10.3389/fnhum.2015.00671

**Published:** 2015-12-22

**Authors:** Kei Saito, Hideaki Onishi, Shota Miyaguchi, Shinichi Kotan, Shuhei Fujimoto

**Affiliations:** ^1^Department of Physical Therapy, Niigata University of Health and WelfareNiigata, Japan; ^2^Institute for Human Movement and Medical Sciences, Niigata University of Health and WelfareNiigata, Japan; ^3^Graduate School of Medicine, Kyoto UniversityKyoto, Japan

**Keywords:** corticospinal excitability, corticospinal pathways, short-latency afferent inhibition, motor cortex, peripheral electrical stimulation

## Abstract

**Objective:** We investigated the transient effect of short-duration paired-pulse electrical stimulation (ppES) on corticospinal excitability and the after-effect of long-duration ppES on excitability, short-latency afferent inhibition (SAI), and afferent facilitation (AF).

**Methods:** A total of 28 healthy subjects participated in two different experiments. In Experiment 1, motor-evoked potentials (MEPs) were measured in the abductor pollicis brevis (APB) and abductor digiti minimi (ADM) muscles before and immediately after short-duration ppES (5 s) at various inter-pulse intervals (2, 3, 4, 5, 6, 7, 10, 15, 20, and 30 ms). In Experiment 2, MEPs, SAI, and AF were measured before, immediately, and 20 and 40 min after long-duration ppES (20 min, inter-pulse interval of 5 and 15 ms) and peripheral electrical stimulation (20 min, 10 and 20 Hz).

**Results:** Short-duration ppES with inter-pulse intervals of 5 and 20 ms significantly increased MEP measured in APB but not in ADM. Long-duration ppES with an inter-pulse interval of 5 ms significantly decreased SAI but not MEPs in APB. In contrast, long-duration ppES did not affect ADM.

**Conclusion:** The afferent inputs induced by ppES-5 ms were effective for transiently increasing MEP and sustaining SAI reduction.

## Introduction

In the sensory motor systems of patients with central nervous system injuries, several studies have shown that afferent input induced by peripheral electrical stimulation (PES) was effective for increasing excitability of the pathway between primary motor cortex (M1) and skeletal muscles (Everaert et al., 2010; Tarkka et al., 2011). This is because of an anatomical connection between the peripheral nerve and M1 via the primary sensory cortex (S1; Jones, 1983). Thus, it is well known that afferent inputs from peripheral nerves have a critical role in modulating corticospinal excitability, including M1. Indeed, many studies using transcranial magnetic stimulation (TMS) reported PES-induced increase in corticospinal excitability (Ridding et al., 2000, 2001; Kaelin-Lang et al., 2002; Khaslavskaia et al., 2002; McKay et al., 2002; Charlton et al., 2003; Knash et al., 2003; Tinazzi et al., 2005; Mang et al., 2010, 2011; Chipchase et al., 2011a,b; Andrews et al., 2013; Saito et al., 2014).

The delivery pattern of afferent inputs from the periphery is important for determining the extent of modulation of cortical excitability. For example, afferent inputs induced by either paired-pulse electrical stimulation (ppES) or paired tactile stimulation, which consisted of two afferent pulses separated by an appropriate delay, inhibited S1 excitability (Ragert et al., 2008; Wühle et al., 2011; Lenz et al., 2012; Young-Bernier et al., 2012; Gatica Tossi et al., 2013). In a previous TMS study, ppES with a short inter-stimulus interval (ISI) between the primary and subsequent pulses inhibited the motor-evoked potential (MEP; Tamè et al., 2015). Thus, ppES has the capacity to modulate the excitability of both S1 and M1. Considering the anatomical connection between peripheral nerves and M1 via S1 (Jones, 1983), afferent inputs induced by ppES might modulate transmission from S1 to M1, as well as S1 and corticospinal excitabilities. Together with the observation that PES was effective for both reducing the activity of inhibitory cortical circuits and enhancing the activity of facilitatory cortical circuits (Mang et al., 2012), similar effects may be obtained with application of ppES.

The ISI between the primary and subsequent electrical pulses is an important factor for the development of modulating cortical excitability. For example, a TMS study reported that MEP inhibition induced by ppES with a long ISI (125 ms) was higher than that induced by ppES with a short ISI (30 ms; Tamè et al., 2015). In an electroencephalogram (EEG) study, ppES with a “short” ISI (>12 ms) was reported to identify the N20m component but not ppES with a “very short” ISI (<9 ms; Hoshiyama and Kakigi, 2002). Considering the evidence that reduction of S1 excitability increased MEP (Jacobs et al., 2014), ppES with a very short ISI might result in an increasing corticospinal excitability more than that with a short ISI.

The location of the stimulus is also a crucial factor for determining the effect of PES. In a TMS study, electrical stimulation of a peripheral nerve increased the corticospinal excitability of the innervated muscle but not non-innervated muscle (Mang et al., 2011). Thus, ppES of peripheral nerves is believed to induce the modulation of corticospinal excitability in only the innervated muscle.

The purpose of this study was to investigate the effect of short-duration ppES with variable ISIs (<30 ms) on corticospinal excitability of both innervated and non-innervated muscles, by recording MEP. Furthermore, we aimed to study the effect of long-duration ppES on modulating MEP and on the activity of cortical circuits that mediate transmission between the periphery and M1, by recording short-latency afferent inhibition (SAI) and afferent facilitation (AF).

## Materials and Methods

### Subjects

A total of 28 neurologically normal right-handed subjects (age range, 20–29 years; mean ± standard deviation, 23.0 ± 3.1 years; three females) participated in this study. All subjects provided written informed consent before participation. This study was performed in accordance with the Declaration of Helsinki and approved by the ethics committee of Niigata University of Health and Welfare (17505-140703).

### Electromyogram Recording

The subjects were comfortably seated in a chair and then his or her right hand was placed on a table with the palm perpendicular to the horizontal plane. Surface electromyograms were recorded using disposable silver–silver chloride surface electrodes (N-00-S; Mets Inc., Tokyo, Japan) placed over abductor pollicis brevis (APB) and abductor digiti minimi (ADM) muscles of the right hand. The signals were amplified (×1000) using an amplification system (A-DL-720-140; 4 Assist; Tokyo, Japan) and sampled at 1 kHz using an A/D converter (PowerLab 8/30; AD Instruments, Colorado Springs, CO, USA).

### Experimental Procedure

#### ppES of the Median Nerve

To the right median nerve, ppES was delivered through a bipolar electrode that was connected to an electrical generator (SEN-7203; Nihon Kohden Co., Tokyo, Japan) through an isolator (SS-104; Nihon Kohden Co.). The stimulus intensity was set to 80% of the motor threshold, which was defined as the lowest stimulation that evoked the M-wave of the APB muscle, This intensity was selected to ensure that the stimulation produced sensory perception without muscle twitch with reference to the work of Tinazzi et al. (2005) that employed approximately 1.5 mA below the motor threshold (85–90% of the motor threshold) as stimulation intensity. In this study, we employed a lower intensity than that reported by Tinazzi et al. (2005) because ppES at 90% of the motor threshold sometimes induced muscle twitch. The inter-train interval was set to 100 ms, and the pulse width was set to 1 ms.

#### Experiment 1: Effect of Short-Duration ppES on MEP

**FIGURE 1:**
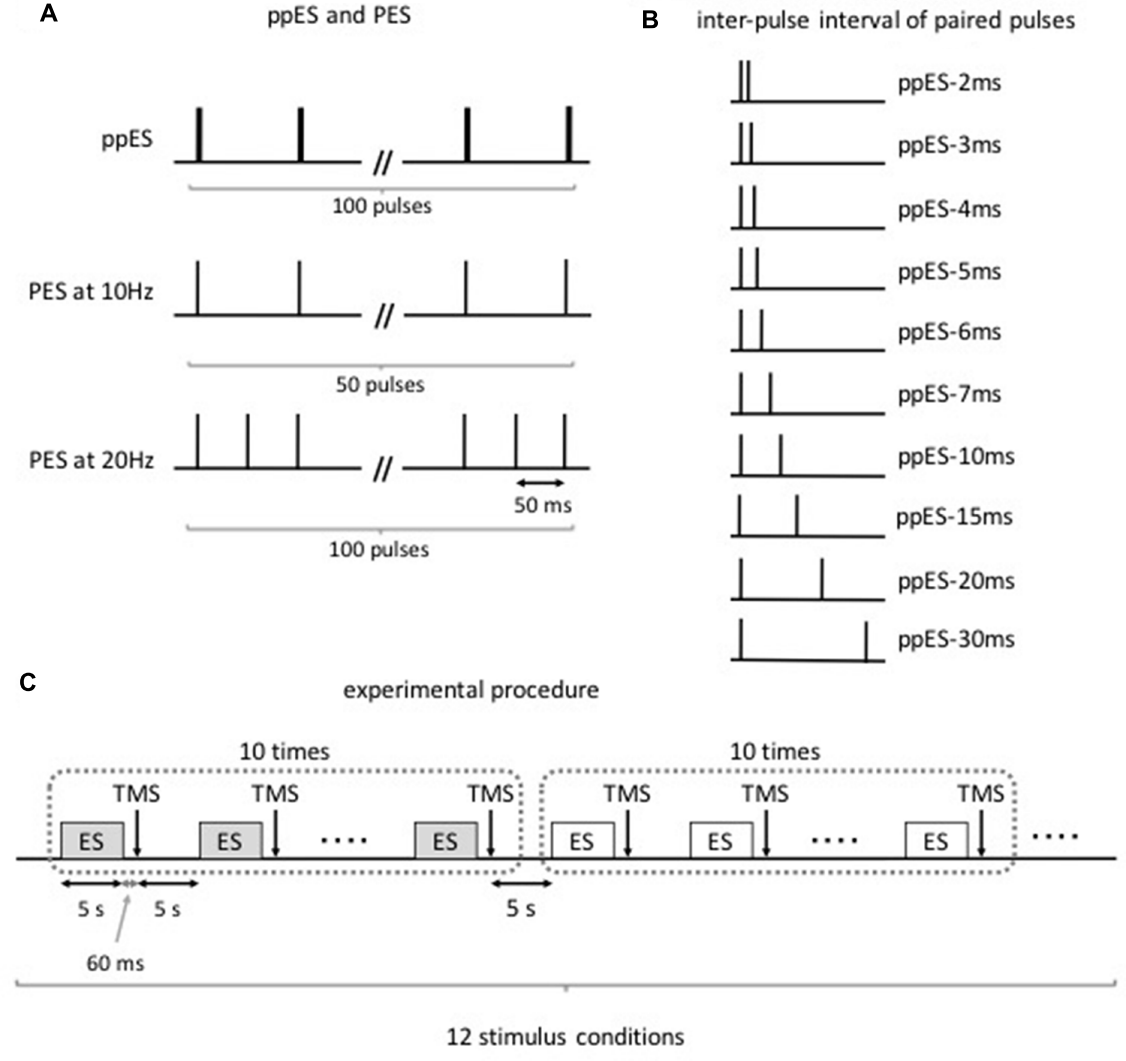
**Paired-pulse electrical stimulation (ppES) and conventional peripheral electrical stimulation (PES) condition (Experiment 1).** **(A)** The ppES and conventional PES protocol. A ppES consisted of two single pulses delivered every 100 ms (total 100 pulses). In contrast, PES consisted of single pulse delivered every 100 ms (total 50 pulses) or 50 ms (total 100 pulses). **(B)** The inter-pulse interval of two single pulses in ppES. The inter-pulse interval between two single pulses was varied (2, 3, 4, 5, 6, 7, 10, 15, 20, 25, and 30 ms). **(C)** Each stimulus condition, including a 5-s stimulus period and 5-s rest period, was continuously performed 10 times. Single TMS pulse was delivered 60 ms after each stimulus period. The stimulus conditions were performed in the following order: ppES-2 ms, ppES-3 ms, ppES-4 ms, ppES-5 ms, ppES-6 ms, ppES-7 ms, ppES-10 ms. ppES-15 ms, ppES-20 ms, ppES-30 ms, PES at 10 Hz, and PES at 20 Hz. The condition order counterbalanced among the subjects using the Latin square.

Twelve subjects were subjected to the following stimulus conditions (**Figures 1A,B**): (i) ppES with a train of two single pulses at various inter-pulse intervals (2, 3, 4, 5, 6, 7, 10, 15, 20, and 30 ms); (ii) PES at 10 Hz (ISI of 100 ms); and (iii) PES at 20 Hz (ISI of 50 ms). These three stimulus conditions were successively performed on the same day. The condition order was counterbalanced among the subjects using the Latin square. The stimulus conditions were performed in the following order: ppES-2 ms, ppES-3 ms, ppES-4 ms, ppES-5 ms, ppES-6 ms, ppES-7 ms, ppES-10 ms. ppES-15 ms, ppES-20 ms, ppES-30 ms, PES at 10 Hz, and PES at 20 Hz. However, the first stimulus condition to be performed differed with respect to the subjects. For example, one subject was subjected to ppES-2 ms condition first and finally PES at 20 Hz condition, while another subject was subjected to ppES-3 ms condition first and finally ppES-2 ms condition. To assess the change in corticospinal excitability caused by ppES or PES, we measured MEP of APB and ADM muscles 60 ms after the end of ppES or PES using TMS.

The experimental procedure is shown in **Figure 1C**. First, all subjects underwent 10–15 trials of a single TMS paradigm to measure their baseline MEP. After baseline measurement, the volunteers were subjected to the stimulus conditions in an assigned order. Each stimulus condition period was continuously performed 10 times. The conditions comprised ppES in which the stimulus duration was set to 5 s, and TMS was measured 60 ms after the last electrical stimulus pulse. Intervals between the stimulus periods and between the stimulus conditions were set to 5 s to minimize the summative effect of electrical stimulation on MEPs. An interval of 60 ms was selected between the last electrical pulse and TMS trigger to minimize the influence of electrical stimulus pulse on MEP and detect the effect of electrical stimulation on MEP with reference to the our previous study (Saito et al., 2014) that an interval of 60 ms between the last electrical pulse and the TMS trigger did not affect the change in MEP induced by a short duration of PES. Ten TMS trials were performed for each ppES or PES condition.

#### Experiment 2: Effect of Long-Duration ppES on MEP, SAI, and AF

This experiment used a double-blind crossover experimental design to compare the effect of long-duration ppES or PES on MEP, SAI, AF, and resting motor threshold. Sixteen volunteers were subjected to the following stimulus conditions: (i) ppES with a train of two single pulses at inter-pulse intervals of 5 ms (ppES-5 ms); (ii) ppES with a train of two single pulses at inter-pulse intervals of 15 ms (ppES-15 ms); (iii) PES at 10 Hz (inter-pulse intervals of 100 ms); and (iv) PES at 20 Hz (inter-pulse intervals of 50 ms). Six of these subjects had participated in Experiment 1. This experiment was performed on a separate day at least 7 days apart from Experiment 1. Inter-pulse intervals of 5 and 15 ms were chosen because Experiment 1 showed that short-duration ppES-5 ms increased MEP the most and short-duration ppES-15 ms did not affect MEP. The experiments using the four stimulus conditions were performed at least 7 days apart. For each condition, 20 min of ppES or PES was applied over the right median nerve at the wrist. The experimenter who performed the TMS measurement as well as the subjects did not know the assigned order.

**FIGURE 2:**
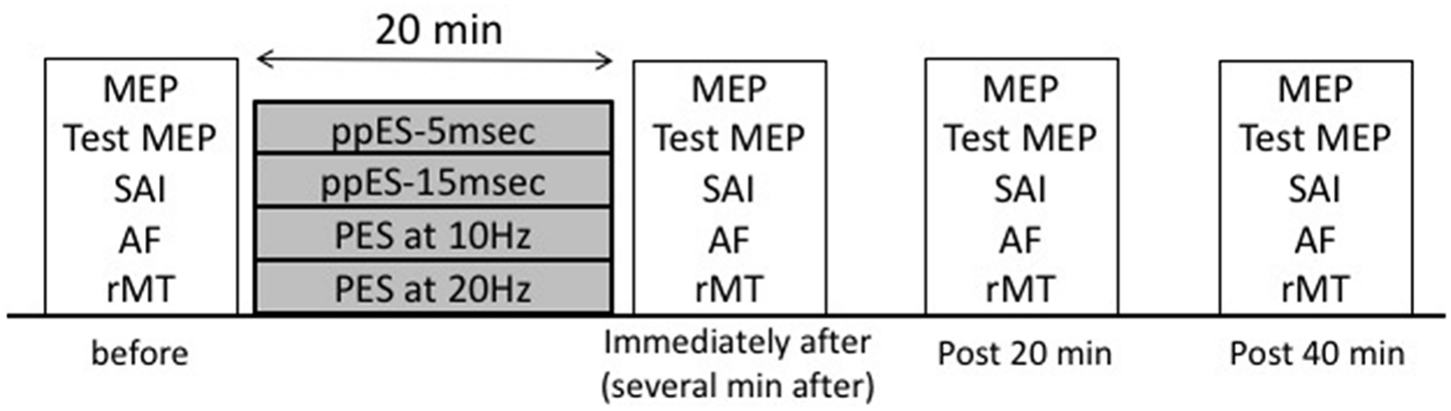
**Experimental protocol (Experiment 2).** Transcranial magnetic stimulation (TMS) measurements [motor-evoked potential (MEP), short-latency afferent inhibition (SAI)], afferent facilitation (AF), and resting motor threshold) were made before, immediately, and 20 and 40 min after the following stimulus conditions: (i) ppES with a train of two single pulses at an inter-pulse interval of 5 ms (ppES-5 ms); (ii) ppES with a train of two single pulses at an inter-pulse interval of 15 ms (ppES-15 ms); (iii) PES at 10 Hz; (iv) PES at 20 Hz. In each stimulus condition, 20 min of ppES or PES was delivered.

The experimental procedure is shown in **Figure 2**. Before starting each condition, the baseline TMS measurements (MEP, SAI, AF, and resting motor threshold) of all the subjects were performed. After the baseline measurements, the subjects were subjected to the ppES or PES conditions in an assigned order. Each condition comprised four TMS measurement blocks (before, immediately, and 20 and 40 min after each ppES or PES). All subjects underwent 20 single TMS trials to measure MEP. After single-pulse TMS measurement, the subjects underwent 20 single-pulse TMS trials to measure the test MEP using adjusted TMS intensity to evoke approximately 1–1.5 mV in APB and 16 paired single-pulse TMS and median nerve electrical stimulation trials to measure SAI and AF in a randomly assigned order in each TMS measurement block (total 52 trials).

### Measurement of MEP

Transcranial magnetic stimulation was applied to the left motor cortex at the optimal site (hot spot) for eliciting an MEP for the right APB muscle using a figure-eight-shaped coil (diameter of 90 mm) connected to a Magstim 200 (Magstim Co. Ltd., Whitland, UK). The hot spot was defined at the site where the largest MEP of APB was elicited by moving the coil around the assumed motor hotspot at a slightly suprathreshold stimulation. The coil was held tangentially to the scalp at an angle of 45° to the mid-sagittal line. The optimal position of the coil was marked on the cap placed on the scalp as a reference and continually checked throughout the experiment. In Experiment 1, TMS intensity was set to 120% of the resting motor threshold. The resting motor threshold was obtained in the APB muscle and defined as the minimum TMS intensity producing a response amplitude > 50 μV in at least five out of 10 trials with the muscles relaxed. In Experiment 2, TMS intensity was set to evoke a MEP of approximately 1 mV in the APB muscle.

### Measurement of SAI and AF

We used a protocol for assessment of SAI and AF that was described in previous reports (Mariorenzi et al., 1991; Deletis et al., 1992; Chen et al., 1999; Tokimura et al., 2000; Roy and Gorassini, 2008; Devanne et al., 2009; Russmann et al., 2009). SAI and AF were examined by applying an electrical stimulus over peripheral nerves immediately before delivering single-pulse TMS over the motor cortex. A test stimulus (TS) was applied to the left motor cortex using a figure eight-shaped coil connected to the TMS, and a conditioning stimulus (CS) was applied to the right median nerve through a bipolar electrode, which was connected to an electrical generator through an isolator. The TS intensity was carefully adjusted to evoke an unconditioned MEP ranging from approximately 1–1.5 mV in APB. TS intensity was adjusted again to approximately 1–1.5 mV for each measurement block. TS intensity was adjusted again after ppES or PES to give similar before and after ppES or PES values. The CS intensity was adjusted to just above the motor threshold in APB. The motor threshold was defined as a small but consistent M-wave. The conditioning-test interval was set to 22 ms for assessment of SAI and 55 ms for assessment of AF (Deletis et al., 1992; Tokimura et al., 2000).

### Data Analysis

In Experiment 1, the peak-to-peak amplitude of MEP was measured and expressed as a percentage of the mean MEP amplitude observed at baseline. The MEP data were analyzed by one-way repeated measured analysis of variance (ANOVA) with the stimulus condition (ppES-2 ms, ppES-3 ms, ppES-4 ms, ppES- 5 ms, ppES-6 ms, ppES-7 ms, ppES-10 ms, ppES-15 ms, ppES-20 ms, and ppES-30 ms) as within-participants factor. If the factor showed a significant main effect, *post hoc* testing was performed by the Tukey multiple comparison test.

In Experiment 2, the peak-to-peak amplitude of MEP was measured in all TMS measurements blocks. To express SAI and AF, the mean MEP amplitude induced by the presence of CS was normalized to mean MEP amplitude induced by the absence of CS (TS alone). The MEP amplitude data were analyzed by two- way repeated ANOVA with time (before, immediately after, and 20 and 40 min after intervention) and stimulus condition (ppES- 5 ms, ppES-15 ms, PES at 10 Hz and PES at 20 Hz). If the factor showed a significant main effect, *post hoc* testing was performed by the Tukey multiple comparison test. With regards to SAI and AF, the SAI and AF data were examined by one-way repeated ANOVA with time (before, immediately after, and 20 and 40 min after intervention) as within-participants factor. If the factor showed a significant main effect, *post hoc* testing was performed by the Tukey multiple comparison test. Furthermore, to compare the effects of ppES and PES on SAI and AF, mean SAI and AF values were measured immediately after and 20 and 40 min after stimulation. The values were averaged and expressed as SAI and AF changes from pre-SAI and AF values, respectively. The SAI and AF changes data were analyzed by a one-way repeated ANOVA with stimulus condition (ppES-5 ms, ppES-15 ms, PES at 10 Hz, and PES at 20 Hz) as within-participants factor. To ensure a stable unconditioned MEP throughout all experiments, unconditioned MEP data evoked by TS alone among each TMS measurement block were compared by two-way repeated ANOVA with time (before, immediately, and 20 and 40 min after intervention) and stimulus condition (ppES-5 ms, ppES-15 ms, PES at 10 Hz and PES at 20 Hz). The data of change in resting motor threshold were analyzed by one-way repeated ANOVA with time (before, immediately, and 20 and 40 min after intervention) as within-participants factor.

All statistical analyses were performed using SPSS 15.0 for Windows. Statistical significance was defined as *p* < 0.05.

## Results

### Effect of Short-Duration ppES on MEP (Experiment 1)

**FIGURE 3:**
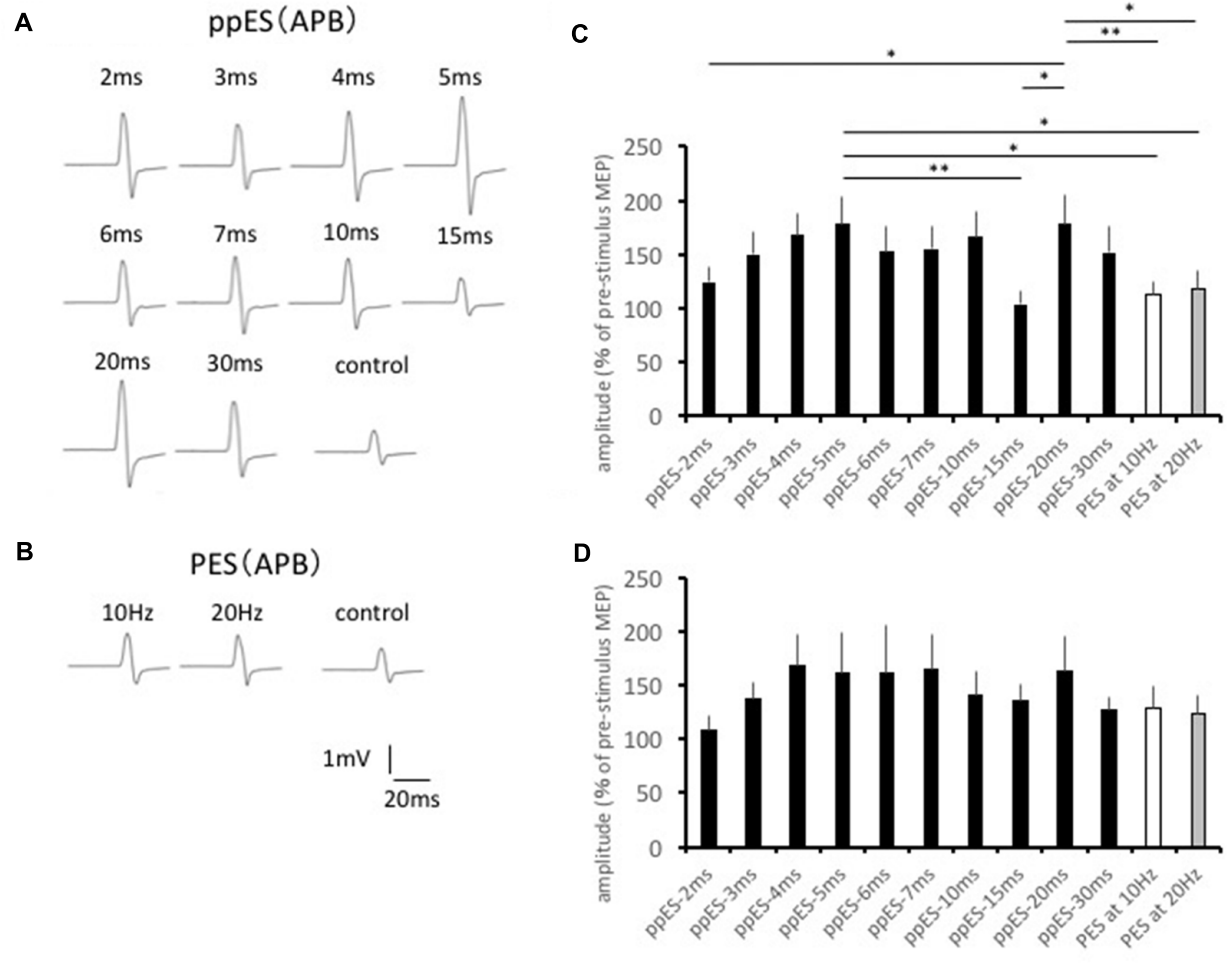
**The effect of short-duration ppES and PES on the MEP recorded in the abductor pollicis brevis (APB) muscle.** **(A)** Typical MEP waveforms recorded in APB muscle in response to ppES. **(B)** Typical MEP waveforms recorded in APB muscle in response to PES. **(C)** The changes in the group mean MEP of APB muscle (*n* = 12) induced by each stimulus condition. The value was expressed as the percentage of baseline MEP measured before the stimulation. One-way-repeated measured ANOVA identified a significant main effect for the stimulus condition on MEP amplitude in APB muscle [*F*(11,121) = 4.175, *p* = 0.000, MSE = 1901.679, η^2^ = 0.28]. A ppES-5 ms was more effective for increasing MEP compared with ppES-20 ms, and PES at 10 Hz and 20 Hz (all *p* < 0.05). ^∗^*p* < 0.05, ^∗∗^*p* < 0.01. Error bars indicate standard error (SE). **(D)** The changes in the group mean MEP of ADM muscle (*n* = 12) induced by each stimulus condition. There was no significant modulation of MEP induced by ppES and PES [*F*(11,121) = 1.822, *p* = 0.057, MSE = 2602.381, η^2^ = 0.14]. Error bars indicate SE.

The typical MEP waveforms in the APB muscle from 1 participant are shown in **Figures 3A,B**, and the effects of ppES and PES on mean MEP amplitudes are shown in **Figure 3C**. One-way repeated measured ANOVA identified a significant main effect for the stimulus condition on MEP amplitude in the APB muscle [*F*(11,121) = 4.175, *p* = 0.000, MSE = 1901.679, η^2^ = 0.28]. *Post hoc* analysis showed that ppES-5 ms was significantly effective in increasing MEP amplitude compared with ppES-15 ms, PES at 10 Hz and PES at 20 Hz (*p* = 0.005, *r* = 0.76 for ppES-15 ms; *p* = 0.018, *r* = 0.71 for PES at 10 Hz; *p* = 0.033, *r* = 0.63 for PES at 20 Hz). Furthermore, ppES-20 ms was also significantly higher compared with ppES-2 ms, ppE-15 ms, PES at 10 Hz and PES at 20 Hz (*p* = 0.043, *r* = 0.55 for ppES-2 ms; *p* = 0.002, *r* = 0.73 for ppES-15 ms; *p* = 0.007, *r* = 0.61 for PES at 10 Hz; *p* = 0.013, *r* = 0.55 for PES at 20 Hz). These results indicated that ppES-5 ms and ppES-20 ms were much more effective in facilitating MEPs measured in APB than ppES-15 ms, PES at 10 Hz and PES at 20 Hz.

The changes induced in MEP in ADM are summarized in **Figure 3D**. One-way repeated-measured ANOVA showed no significant differences in MEP waveforms between the stimulus conditions in the ADM muscle [*F*(11,121) = 1.822, *p* = 0.057, MSE = 2602.381, η^2^ = 0.14], which indicated the similar effect of ppES on MEP to PES in ADM.

### Effect of Long-Duration ppES and PES on MEP (Experiment 2)

**FIGURE 4:**
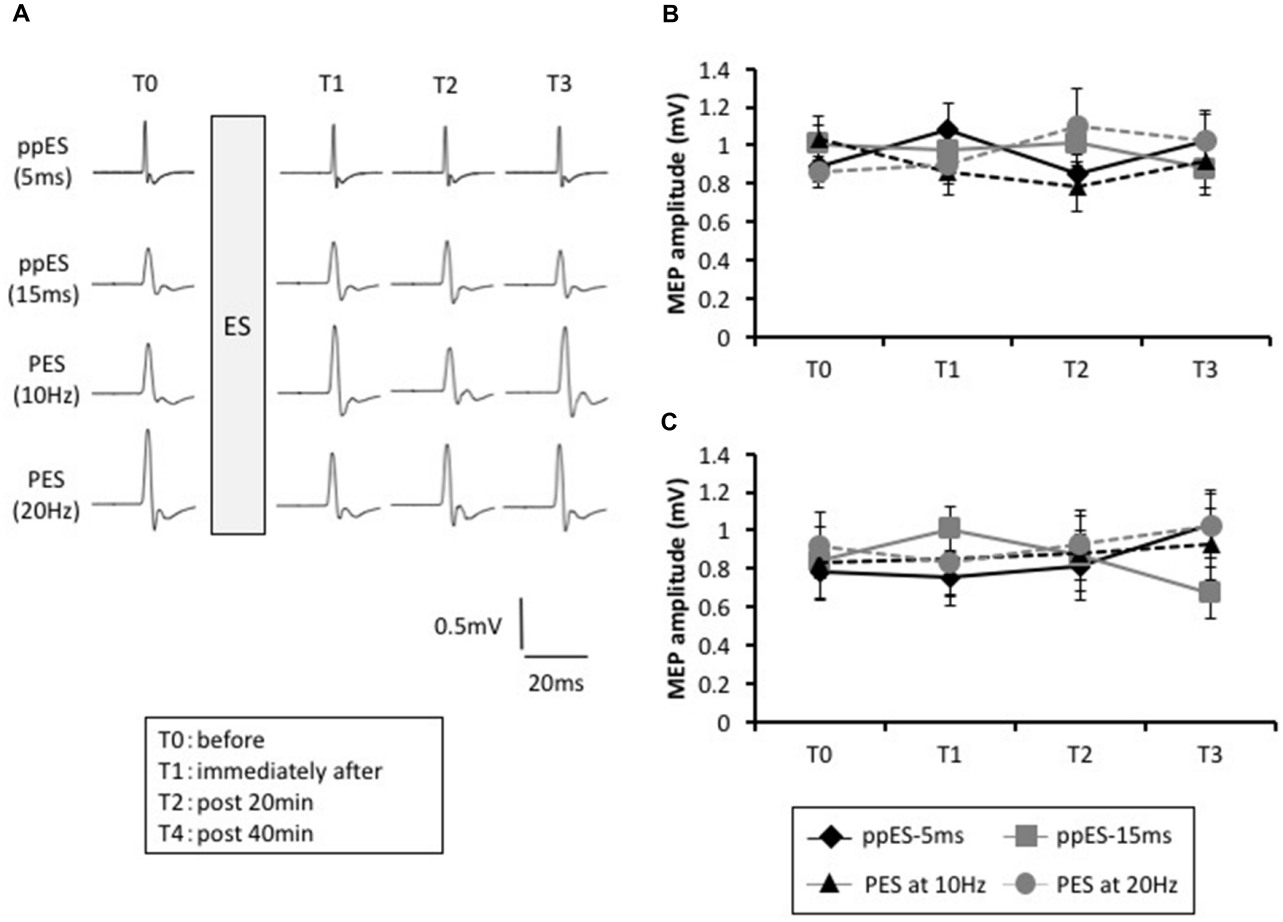
**The effect of short-duration ppES and PES on the MEP recorded in the abduction digiti minimi (ADM) muscle.** **(A)** Typical MEP waveforms recorded in APB muscle in response to ppES, and PES at 10 Hz and 20 Hz for each time (pre, immediately, and 20 and 40 min after electrical stimulation). **(B)** The changes in the group mean MEP amplitude of APB muscle (*n* = 12) induced by each stimulus condition. There was no significant effect of stimulus condition [*F*(1.823,27.342) = 0.022, *p* = 0.972] or time [*F*(3,45) = 0.291, *p* = 0.883] on MEP amplitude. Furthermore, no significant interaction was identified between stimulus condition and time [*F*(4.252,63.787) = 2.274, *p* = 0.067]. Error bars indicate SE. **(C)** The changes in the group mean MEP amplitude of ADM muscle (*n* = 12) induced by each stimulus condition. There was no significant effect of stimulus condition [*F*(2.192,32.886) = 0.410, *p* = 0.691] or time [*F*(3,45) = 0.204, *p* = 0.893] and no interaction between stimulus condition and time [*F*(4.432,66.481) = 2.097, *p* = 0.084]. Error bars indicate SE.

The typical MEP waveforms in the APB muscle from a representative participant are shown in **Figure 4A**, and the MEP changes induced by ppES and PES are shown in **Figure 4B**. Two-way repeated ANOVA did not identify a significant effect of stimulus condition [*F*(3,45) = 0.219, *p* = 0.883, MSE = 0.334, η^2^ = 0.01] or time [*F*(1.823,27.342) = 0.022, *p* = 0.972, η^2^ = 0.00] on MEP amplitude. Furthermore, no significant interaction was identified between stimulus condition and time [*F*(4.252,12.306) = 2.274, *p* = 0.067, MSE = 0.193, η^2^ = 0.05].

The MEP changes in ADM are summarized in **Figure 4C**. Two-way repeated ANOVA revealed that there was no significant effect of stimulus condition [*F*(3,45) = 0.204, *p* = 0.893, MSE = 0.421, η^2^ = 0.01] or time [*F*(3,45) = 0.401, *p* = 0.753, MES = 0.135, η^2^ = 0.01] and no interaction between stimulus condition and time [*F*(4.432,66.481) = 2.097, *p* = 0.084, MSE = 0.201, η^2^ = 0.05].

These results indicated that application of long-duration ppES and PES on the median nerve did not affect MEPs measured in APB and ADM.

### Effect of Long-Duration ppES and PES on SAI and AF (Experiment 2)

**FIGURE 5:**
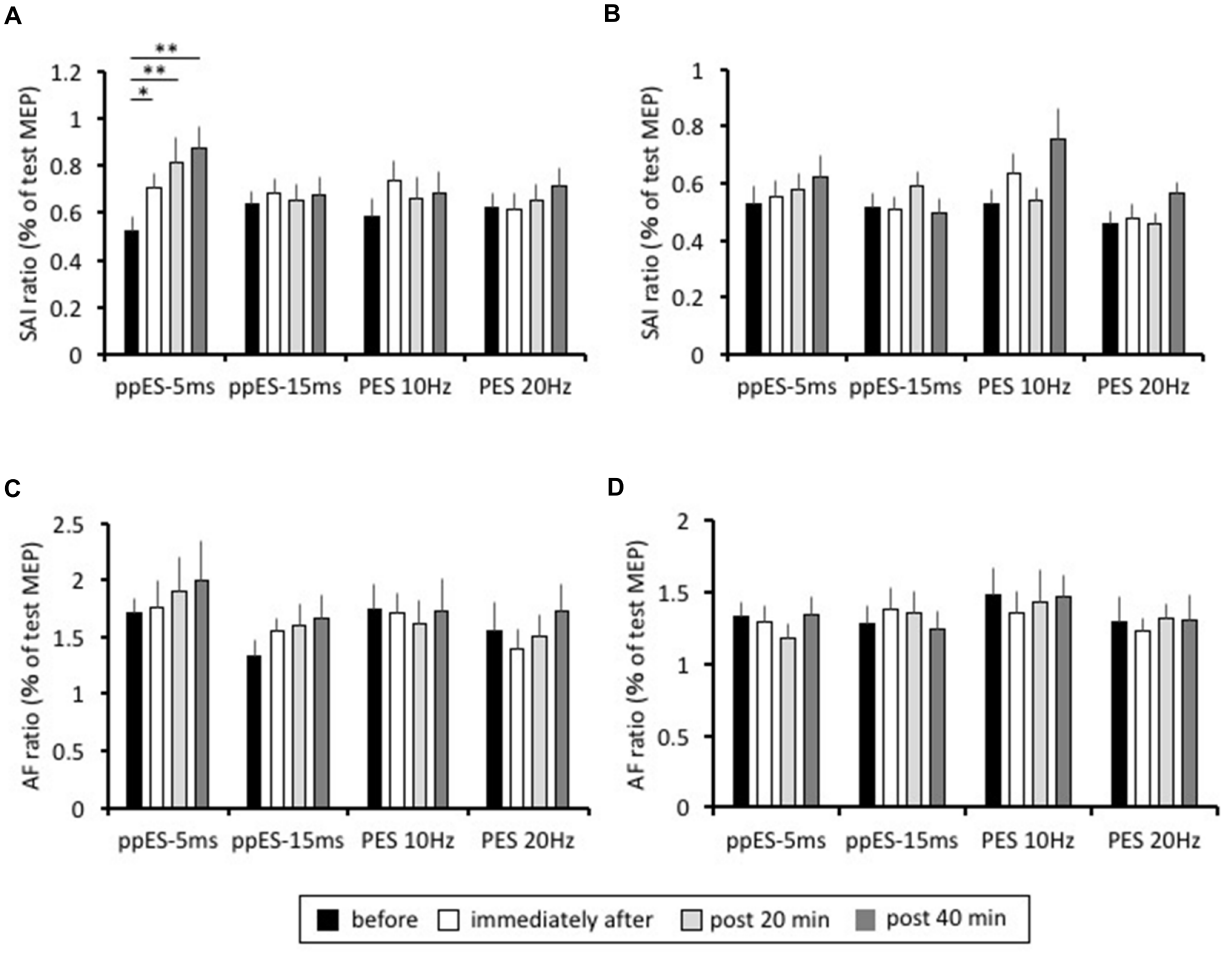
**The effect of long-duration ppES and PES on the SAI and AF recorded in the APB and ADM muscle.** **(A)** The group mean SAI of APB muscle (*n* = 12) induced by each stimulus condition. The SAI value was expressed as percentage of unconditioned MEP amplitude. In application of long-duration ppES-5 ms, SAI was significantly reduced immediately after (*p* = 0.047), at 20 min after (*p* = 0.000) and at 40 min after (*p* = 0.000) relative to the pre-SAI value. **(B)** The group mean SAI of ADM muscle (*n* = 12) induced by each stimulus condition. The SAI value was expressed as percentage of unconditioned MEP amplitude. There was no significant modulation of SAI induced by ppES and PES (all *p* > 0.05). **(C)** The group mean AF of APB muscle (*n* = 12) induced by each stimulus condition. The AF value was expressed as percentage of unconditioned MEP amplitude. There was no significant modulation of AF induced by ppES and PES (all *p* > 0.05). **(D)** The group mean AF of ADM muscle (*n* = 12) induced by each stimulus condition. The AF value was expressed as percentage of unconditioned MEP amplitude. There was no significant modulation of AF induced by ppES and PES (all *p* > 0.05). ^∗^*p* < 0.05, ^∗∗^*p* < 0.01. Error bars indicate SE.

The lasting changes induced in SAI are summarized in **Figure 5A**. In application of long-duration ppES-5 ms, one-way repeated ANOVA revealed that there was a significant effect of time on the SAI recorded in the APB muscle [*F*(3,45) = 10.394, *p* = 0.000, MSE = 0.035, η^2^ = 0.41]. *Post hoc* analysis showed that SAI was significantly reduced immediately after (*p* = 0.047, *r* = 0.71), at 20 min after (*p* = 0.000, *r* = 0.68), and at 40 min after (*p* = 0.000, *r* = 0.8) relative to the pre-SAI value. Conversely, one-way repeated ANOVA showed that SAI recorded in APB was relatively constant with time in application of ppES-20 ms, PES at 10 Hz and PES at 20 Hz [*F*(3,45) = 0.148, *p* = 0.930, MSE = 0.052, η^2^ = 0.01 for ppES-15 ms; *F*(3,45) = 0.976, *p* = 0.412, MSE = 0.061, η^2^ = 0.06 for PES at 10 Hz; *F*(3,45) = 1.142, *p* = 0.342, MSE = 0.032, η^2^ = 0.07 for PES at 20 Hz]. Furthermore, one-way repeated ANOVA revealed that there was a significant effect of stimulus condition on the SAI changes from pre-SAI value in the APB muscle [*F*(3,45) = 3.786, *p* = 0.017, MSE = 0.051, η^2^ = 0.2]. *Post hoc* analysis represented that SAI change from pre-SAI induced by ppES-5 ms was significantly higher than that by ppES-15 ms and PES at 20 Hz [ppES-15 ms; *p* = 0.022, *r* = 0.66, PES at 10 Hz; *p* = 0.182, *r* = 0.39, PES at 20 Hz; *p* = 0.027, *r* = 0.66], indicating that ppES-5 ms was significantly effective in decreasing SAI compared to ppES-15 ms and PES at 20 Hz.

In the ADM muscle, the SAI values recorded at each measurement block are shown in **Figure 5B**. One-way repeated ANOVA revealed no significant effect of time on the degree of SAI recorded for all stimulus conditions [*F*(3,45) = 0.696, *p* = 0.559, MSE = 0.034, η^2^ = 0.05 for ppES-5 ms; *F*(3,45) = 1.366, *p* = 0.265, MSE = 0.022, η^2^ = 0.08 for ppES-15 ms; *F*(3,45) = 2.765, *p* = 0.053, MSE = 0.064, η^2^ = 0.16 for PES at 10 Hz; *F*(3,45) = 2.701, *p* = 0.057, MSE = 0.015, η^2^ = 0.15 for PES at 20 Hz]. Furthermore, one-way repeated ANOVA showed that there was no significant effect of stimulus condition on the SAI changes from pre-SAI value [*F*(3,45) = 0.687, *p* = 0.565, MSE = 0.042, η^2^ = 0.04].

The AF value recorded in APB at each measurement block is summarized in **Figure 5C**. One-way repeated ANOVA did not identify a significant effect of time on AF recorded in APB [*F*(3,45) = 0.904, *p* = 0.447, MSE = 0.306, η^2^ = 0.06 for ppES-5 ms; *F*(3,45) = 2.622, *p* = 0.062, MSE = 0.132, η^2^ = 0.15 for ppES-15 ms; *F*(3,45) = 0.215, *p* = 0.886, MSE = 0.242, η^2^ = 0.02 for PES at 10 Hz; *F*(3,45) = 1.688, *p* = 0.183, MSE = 0.142, η^2^ = 0.13 for PES at 20 Hz]. One-way repeated ANOVA did not identify significant effect of stimulus condition on AF changes from pre-AF value [*F*(3,45) = 1.038, *p* = 0.385, MSE = 0.385, η^2^ = 0.07].

The AF value recorded in ADM at each measurement block were represented in **Figure 5D**. One-way repeated ANOVA showed that there was no significant effect of time on AF recorded in ADM nor was there an effect of [*F*(3,45) = 1.172, *p* = 0.331, MSE = 0.082, η^2^ = 0.07 for ppES-5 ms; *F*(3,45) = 0.726, *p* = 0.542, MSE = 0.094, η^2^ = 0.05 for ppES-15 ms; *F*(3,45) = 0.239, *p* = 0.869, MSE = 0.224, η^2^ = 0.02 for PES at 10 Hz; *F*(3,45) = 0.187, *p* = 0.905, MSE = 0.121, η^2^ = 0.01 for PES at 20 Hz] One-way repeated ANOVA did not identify significant effect of stimulus condition upon AF changes recorded in ADM [*F*(3,45) = 0.127, *p* = 0.943, MSE = 0.289, η^2^ = 0.01]. The average MEP evoked in either APB or ADM muscles by TS alone showed no significant effect related to the type of stimulus condition [*F*(2.084,31.263) = 1.284, *p* = 0.292, MSE = 0.522, η^2^ = 0.05 for APB; *F*(3,45) = 1.188, *p* = 0.325, MSE = 0.365, η^2^ = 0.04 for ADM] or of time [*F*(3,45) = 0.21, *p* = 0.889, MSE = 0.053, η^2^ = 0.00 for APB; *F*(1.481, 22.219) = 2.919, *p* = 0.088, MSE = 0.233, η^2^ = 0.03 for ADM] on the test MEP amplitude (**Table 1**). Furthermore, the analysis revealed no significant interaction between the stimulus condition and time for the ADM muscle, but there was a significant interaction between the stimulus condition and time in the APB muscle. These results indicated that MEP amplitude evoked by TS was well-adjusted.

**Table 1:** Motor-evoked potential (MEP) amplitude evoked by test stimulation at each time.

	APB muscle				ADM muscle			
		
	Before	Immediately after	Post 20 min	Post 40 min	Before	Immediately after	Post 20 min	Post 40 min
ppKS-5 ms	0.84 ± 0.08	0.84 ± 0.08	0.94 ± 0.08	0.82 ± 0.08	0.78 ± 0.16	0.79 ± 0.15	0.94 ± 0.21	1.02 ± 0.23
ppES-15 ms	1.12 ± 0.15	1.10 ± 0.14	0.98 ± 0.11	0.96 ± 0.13	0.90 ± 0.18	0.99 ± 0.19	0.84 ± 0.18	0.89 ± 0.17
PES at 10Hz	0.91 ± 0.10	0.89 ± 0.14	0.99 ± 0.15	1.10 ± 0.14	0.74 ± 0.16	0.83 ± 0.14	0.84 ± 0.17	1.01 ± 0.22
PhS at 20Hz	1.04 ± 0.10	1.08 ± 0.11	1.05 ± 0.10	0.93 ± 0.09	0.99 ± 0.15	1.01 ± 0.16	1.05 ± 0.22	1.13 ± 0.23


*Average **±** SE.*

The SAI and AF results indicated that application of ppES-5 ms on the median nerve had long lasting inhibitory effect of SAI measured in APB, and the effect of ppES-5 ms was higher compared with other stimulus conditions.

### Effect of Long-Duration ppES and PES on Resting Motor Threshold (Experiment 2)

The effects of ppES and PES on resting motor threshold are shown in **Table 2**. One-way repeated ANOVA showed no significant time effect on the resting motor threshold for all stimulus conditions [*F*(3,45) = 1.846, *p* = 0.152, MSE = 2.325, η^2^ = 0.11 for ppES-5 ms; *F*(3,45) = 0.694, *p* = 0.561, MSE = 1.524, η^2^ = 0.05 for ppES-15 ms; *F*(3,45) = 0.807, *p* = 0.497, MSE = 2.241, η^2^ = 0.05 for PES at 10 Hz; *F*(3,45) = 0.371, *p* = 0.774, MSE = 1.236, η^2^ = 0.03 for PES at 20 Hz].

**Table 2:** Resting motor threshold of transcranial magnetic stimulation at each time.

	Resting motor threshold			
	
	Before	Immediately after	Post 20 min	Post 40 min
ppKS-5 ms	43.1 ± 1.4	43.4 ± 1.4	43.4 ± 1.4	42.3 ± 1.4
ppES-15 ms	43.5 ± 1.5	44.1 ± 1.7	43.8 ± 1.6	43.4 ± 1.7
PES at 10Hz	43.6 ± l.5	43.4 ± 1.7	44.0 ± 1.9	44.4 ± 1.7
PhS at 20Hz	43.3 ± 1.3	43.2 ± 1.3	43.3 ± 1.3	43.4 ± 1.3


*Average ± SE.*

## Discussion

There were two main findings in this study. First, short-duration ppES using an inter-pulse interval of 5 ms increased corticospinal excitability. Second, long-duration ppES-5 ms decreased activity of the inhibitory circuit that mediates transmission between peripheral nerve and M1 but did not decrease activity of the excitatory circuit or decrease corticospinal excitability.

### Effect of Short-Duration ppES on MEP

We found that short-duration ppES-5 ms increased corticospinal excitability more effectively than did ppES-15 ms and PES at 10 and 20 Hz. Facilitation of corticospinal excitability by PES has been reported in previous PES studies (Ridding et al., 2000, 2001; Kaelin-Lang et al., 2002; Khaslavskaia et al., 2002; McKay et al., 2002; Charlton et al., 2003; Knash et al., 2003; Tinazzi et al., 2005; Mang et al., 2010, 2011; Chipchase et al., 2011a,b; Golaszewski et al., 2012; Andrews et al., 2013; Saito et al., 2014). In particular, our previous study using similar experimental protocols revealed that short-duration PES increased MEP (Saito et al., 2014). The present study revealed that short-duration ppES was more effective for modulation of corticospinal excitability than was short-duration PES. This finding may explain the greater inhibitory effect of short-duration ppES on excitability of S1 than that of short-duration PES. In a previous study, S1 was reported to have an important role in modulating corticospinal excitability (Jacobs et al., 2014) which also found that decreased S1 activity caused by continuous theta-burst stimulation (cTBS) increased MEP. Furthermore, Hoshiyama and Kakigi (2002) revealed that a single electrical pulse could induce N20m as an indicator of S1 excitability, but N20m was not identified using paired electrical pulses at inter-pulse intervals of <9 ms. These results indicated that S1 excitability could be reduced more by this paired electrical pulse condition than by a single electrical pulse. Considering that ppES used in the present study comprised a train of two single electrical pulses, short-duration ppES-5 ms may reduce the excitability of S1 and increase corticospinal excitability.

### Effect of Long-Duration ppES on MEP

Facilitation of corticospinal excitability by PES has previously been reported (Ridding et al., 2000, 2001; Kaelin-Lang et al., 2002; Khaslavskaia et al., 2002; McKay et al., 2002; Charlton et al., 2003; Knash et al., 2003; Tinazzi et al., 2005; Mang et al., 2010, 2011; Chipchase et al., 2011a,b; Golaszewski et al., 2012; Andrews et al., 2013; Saito et al., 2014). Furthermore, we found that short-duration ppES-5 ms was effective in increasing MEP in Experiment 1 of this study. However, in Experiment 2, we found that long-duration ppES did not affect corticospinal excitability. This discrepancy may be due to transient increases in excitability of interneurons connecting pyramidal cells and/or pyramidal cells in M1 by ppES but not by the absence of plastic changes in these cells. In a previous study, PES duration was reported to have an important role in inducing cortical plasticity (McKay et al., 2002; Charlton et al., 2003; Veldman et al., 2014). For example, Veldman et al. (2014) showed that PES for 60–120 min was the most effective condition for inducing cortical plasticity. Thus, the 20 min of ppES used in the present study may be insufficient to induce plastic changes of interneurons connecting pyramidal cells and/or pyramidal cells in M1, even if ppES had a more powerful effect on corticospinal excitability than did PES. Therefore, the time until the start of measuring MEP may be important. In Experiment 1, MEP measurement was performed 60 ms after the end of short-duration ppES, whereas in Experiment 2, the measurement was performed a few minutes after the end of long-duration ppES. Thus, the effect of long-duration ppES may disappear by the start of measuring MEP after long-duration ppES. Conversely, the absence of an effect of long-duration ppES on MEP may be because of insufficient stimulus intensity. In a previous study, PES intensity was reported to have an important role in modulating corticospinal excitability (Chipchase et al., 2011a). That study showed that PES above the motor threshold was more effective for increasing corticospinal excitability than was PES above the sensory threshold. Furthermore, Veldman et al. (2014) reported that somatosensory stimulation decreased corticospinal excitability when PES duration was set to <30 min. Considering ppES intensity (below the motor threshold) and the duration (20 min) used in the current study, long-duration ppES may not be sufficient for increasing corticospinal excitability.

### Effect of Long-Duration ppES on SAI

Attenuation of SAI by 40 min of PES has been revealed in a prior TMS study (Mang et al., 2012) where the decreased activity of inhibitory cortical circuits was detected immediately after electrical stimulation to the peripheral nerve. Similar results were obtained in the present study where it was shown that the application of electrical stimulation to a peripheral nerve induced reduction of SAI. The difference between our study and the work of Mang et al. (2012) was in the duration of an after-effect of electrical stimulation. In their study, PES resulted in transient reduction of SAI. In contrast, we found that ppES with 5 ms ISI reduced SAI for at least 40 min after stimulation. This difference may be responsible for the change of S1 excitability, because it has previously been shown that a change in S1 excitability was related to a reduction of SAI in non-invasive brain stimulation studies (Tsang et al., 2014; Kojima et al., 2015). For example, cTBS applied over S1 was reported to result in reduction of SAI (Tsang et al., 2014). In that study, the sustained reduction of SAI was induced by cTBS, which was effective for a long-lasting decrease in the P25/N33 SEP component, over S1. These results suggested an important role of S1 excitability on the modulation of SAI. In addition, previous SEP studies revealed that a paired median nerve stimulation (MNS) induced a significant reduction in the N20m component, relative to a single MNS, when the ISI was 5 ms (Hoshiyama and Kakigi, 2002). According to the abovementioned evidence, it seems that ppES-5 ms was more effective for decreasing S1 excitability than conventional PES and that SAI was consequently disinhibited.

### Methodological Considerations

The change in spinal interneuron excitability and/or the excitability of M1 is likely to be involved in the change in corticospinal excitability. Thus, measurement of the H-reflex amplitude is needed to investigate the mechanism underlying the modulatory effect of the corticospinal excitability by ppES. However, a previous study showed that PES had no effect on modulating the excitability of the spinal interneurons (Thompson et al., 2006), which indicated that the excitability of M1 may be involved in changes of the corticospinal excitability by PES. However, further examination of the mechanism underlying the changes in corticospinal excitability induced by ppES is required.

## Conclusion

We found that short-duration ppES using a specific inter-pulse interval transiently increased corticospinal excitability in the APB. Furthermore, we showed that long-duration ppES decreased SAI in APB, and the effect persisted for at least 40 min after the intervention. Considering the observation that reduced SAI was reported to have an important role in facilitating motor recovery in stroke patients (Di Lazzaro et al., 2012), ppES-5 ms may be useful in rehabilitation of stroke patients with motor paralysis.

## Conflict of Interest Statement

The authors declare that the research was conducted in the absence of any commercial or financial relationships that could be construed as a potential conflict of interest.
